# Brain proton magnetic resonance spectroscopy findings in a Beagle dog with genetically confirmed Lafora disease

**DOI:** 10.1111/jvim.15799

**Published:** 2020-05-17

**Authors:** Neringa Alisauskaite, Katrin Beckmann, Matthias Dennler, Niklaus Zölch

**Affiliations:** ^1^ Neurology Service, Department of Small Animal Surgery, Vetsuisse Faculty University of Zurich Zurich Switzerland; ^2^ Clinic for Diagnostic Imaging, Department of Diagnostics and Clinical Services, Vetsuisse Faculty University of Zurich Zurich Switzerland; ^3^ Department of Forensic Medicine and Imaging Institute of Forensic Medicine, University of Zurich Zurich Switzerland

**Keywords:** canine Lafora diesease, cerebral, genetic disease, metabolic brain disease, myoclonus epilepsy, neurology

## Abstract

Cortical atrophy has been identified using magnetic resonance imaging (MRI) in humans and dogs with Lafora disease (LD). In humans, proton magnetic resonance spectroscopy (1HMRS) of the brain indicates decreased *N*‐acetyl‐aspartate (NAA) relative to other brain metabolites. Brain 1HMRS findings in dogs with LD are lacking. A 6‐year‐old female Beagle was presented with a history of a single generalized tonic‐clonic seizure and episodic reflex myoclonus. Clinical, hematological, and neurological examination findings and 3‐Tesla MRI of the brain were unremarkable. Brain 1HMRS with voxel positioning in the thalamus was performed in the affected Beagle. It identified decreased amounts of NAA, glutamate‐glutamine complex, and increased total choline and phosphoethanolamine relative to water and total creatine compared with the reference range in healthy control Beagles. A subsequent genetic test confirmed LD. Abnormalities in 1HMRS despite lack of changes with conventional MRI were identified in a dog with LD.

**Abbreviations**
1HMRS: proton magnetic resonance spectroscopy
FWHM: full width at half maximum
Glx: glutamate and glutamine complex
Gly: glycine
GPC: glycerophosphocholine
HE: hepatic encephalopathy
LD: Lafora disease
mI: myo‐inositol
MRI: magnetic resonance imaging
NAA: *N*‐acetyl‐aspartate
PE: phosphoethanolamine
SNR: signal‐to‐noise ratio
T1W: T1‐weighted
T2W: T2‐weighted
tCho: total choline
tCr: total creatine
TE: echo time
TR: repetition time

## 1 INTRODUCTION

Lafora disease is a genetic disease with autosomal recessive inheritance affecting people and numerous animal species, including dogs.^1^, ^2^, ^3^, ^4^Certain dog breeds, such as miniature wire‐haired Dachshunds, Basset Hounds, and Beagles are reported to be affected.^3^, ^4^ The disease is characterized by abnormal accumulation of polyglucosan inclusions (also called Lafora bodies), mainly in neurons. To a lesser extent, Lafora bodies also can be found in other cells of the central nervous system and in cells of other organs.^1^, ^5^ This accumulation leads to neurological dysfunction, a classical sign being myoclonus epilepsy.^1^, ^2^ A mutation of the NHLRC1 gene has been identified in affected dogs, and a genetic test is available.^3^, ^4^ Conventional magnetic resonance imaging (MRI) in humans and dogs can be normal or may disclose gray matter atrophy in the brain.^1^, ^2^

Proton magnetic resonance spectroscopy (1HMRS) is a diagnostic imaging tool that assesses the concentration of brain metabolites and is used to characterize several systemic and cerebral diseases in humans and dogs.^6^, ^7^, ^8^, ^9^, ^10^, ^11^, ^12^ Commonly measured brain metabolites include total choline (tCho), which is involved in cell membrane synthesis, *N*‐acetyl‐aspartate (NAA), a neuronal marker, total creatine (tCre), which is responsible for intracellular energy states and myo‐Inositol (mI), a glial cell marker.^6^, ^12^, ^13^ In Lafora disease in humans, 1HMRS identified decreased NAA ratios to tCr, tCho, and mI in cortical, cerebellar, and basal ganglia areas of the brain affected by LD.^14^, ^15^, ^16^ Proton magnetic resonance spectroscopy has not been performed previously in dogs with LD.

### 1.1 Case presentation

A client‐owned 6‐year‐old, 13‐kg, female neutered Beagle was presented to the neurology service in the small animal clinic of the University of Zurich. The dog had a history of 1 generalized tonic‐clonic seizure and multiple episodes of myoclonus seizures, which usually were precipitated by auditory or visual triggers. Blood biochemistry and hematology were performed by the referring veterinarian, including fasted and postprandial bile acid concentrations, and results were within the reference range. The dog was treated with 2 mg/kg phenobarbital PO q12h for 1 month before presentation at the University of Zurich and the blood phenobarbital concentration was 14.9 mg/L. Clinical and neurological examinations did not identify any abnormalities. The neuroanatomical localization was the forebrain. Based on the signalment and seizure semiology, LD was strongly suspected. Blood samples were obtained and genetic testing for the NHLRC1 gene mutation was performed in a commercial laboratory using PCR as described previously.^3^ The dog was sedated with IV midazolam (0.2 mg/kg) and butorphanol (0.2 mg/kg), and anesthesia was induced with IV propofol (2.5 mg/kg) and maintained with sevoflurane gas. During anesthesia the dog received Ringer's actetate (3 mL/kg/h).

Magnetic resonance imaging of the brain was performed with a high‐field 3‐Tesla MRI scanner (Philips Ingenia, Philips AG, Switzerland) equipped with a head/neck/spine coil. The dog was positioned in dorsal recumbency. Studies included gradient echo pre‐ and postcontrast 3D T1‐weighted (T1W) images (echo time [TE] = 4.1 ms, repetition time [TR] = 8.9 ms; slice thickness = 0.6 mm), spin echo 3D T2‐weighted (T2W) images (TE = 180.9 ms, TR = 2300 ms, slice thickness = 0.7 mm) and transverse fluid attenuation inversion recovery (TE = 125 ms, TR = 11 000 ms, slice thickness = 2.5 mm) images. Gadopentetate dimeglumine (0.1 mmol/kg) was administered IV for postcontrast image acquisitions.

Proton magnetic resonance spectroscopy was performed before contrast media administration. The 1HMRS protocol used was the same as previously described with voxel positioning in the left thalamic area (TE = 32 ms, TR = 2000 ms, 240 signal averages).^17^ Proton magnetic resonance spectroscopy data were analyzed using the LCModel software, which fits the spectra as a linear combination of model spectra of metabolites presumably present in the tissue. Simulated spectra of 20 metabolites (alanine, aspartate, glucose, creatine, phosphocreatine, glutamine, glutamate, glycerophosphocholine [GPC], phosphocholine, lactate, mI, NAA, *N*‐acetyl‐aspartyl‐glutamate, scyllo‐inositol, glutathione, taurine, glycine [Gly], phosphoethanolamine [PE], ascorbate, and γ‐aminobutyric acid) were used. Contributions from lipids and macromolecules were simulated in the LCModel. Estimates of the mmol/L concentrations of the metabolites were calculated with the unsuppressed water signal as reference (TE = 32 ms, TR = 2 seconds), estimating a pure gray mater water concentration of 43 300 mmol/L (LCModel setting: WCONC = 43 300) and correcting for relaxation attenuation by an factor of 0.7 (ATTH2O = 0.7). In addition, the metabolite ratios to total creatine (tCr, the sum of creatine and phosphocreatine) were calculated.

A cisternal cerebrospinal fluid collection was performed under aseptic conditions at the end of the MRI investigation.

**FIGURE 1:**
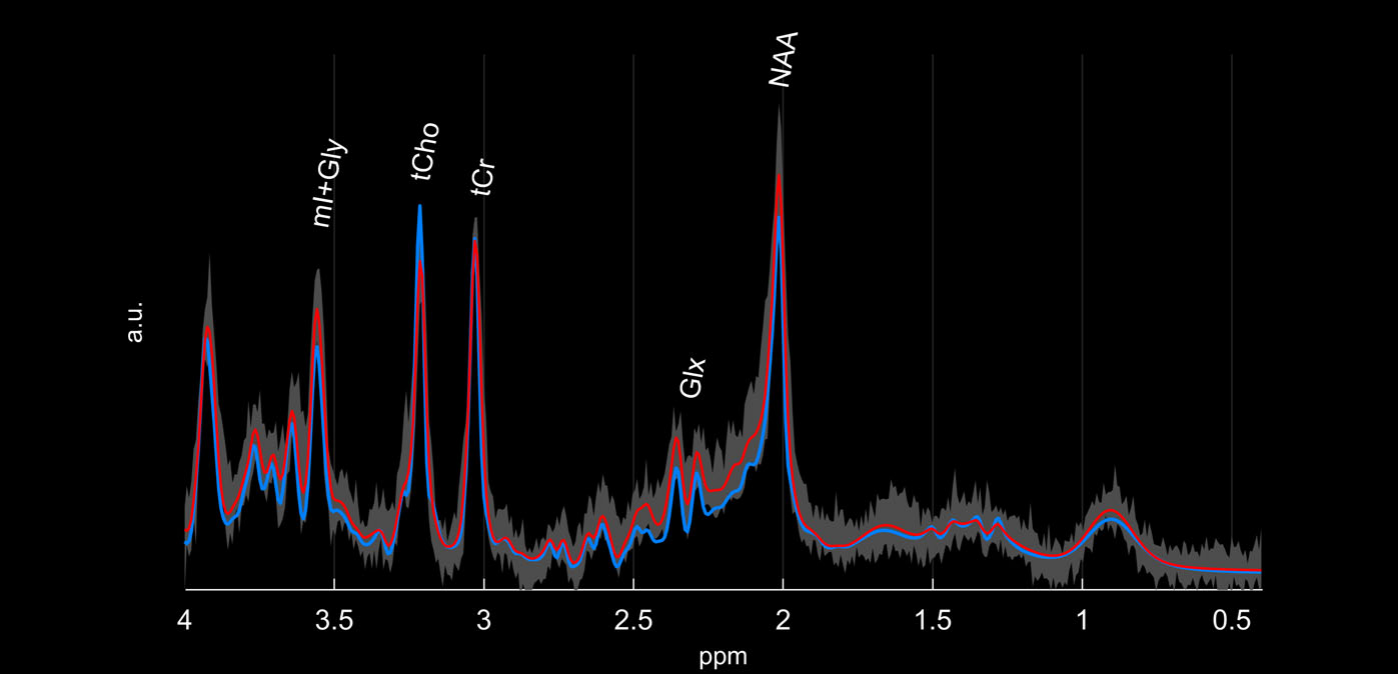
Comparison of the fitted spectrum obtained from the Beagle with LD (light blue line) and the average fitted spectrum of the control Beagles (red line). Highlighted in gray, the entire range of measured values of all 10 healthy Beagles is shown. The NAA and Glx peaks are lower, and the tCho peak is higher in the Beagle with LD compared to control Beagles. Relevant metabolite peaks are marked in the figure. All spectra are scaled with the maximum of the fitted tCr signal for viewing purposes. Glx, glutamate‐glutamine complex; LD, Lafora disease; NAA, *N*‐acetyl‐aspartate; tCho, total choline; tCr, total creatine

In addition, MRI and 1HMRS with voxel positioning in the thalamic area of 12 healthy 3‐ to 6‐year‐old Beagles (with the same acquisition and anesthesia protocol with butorphanol, propofol, and sevoflurane) and archived from an independent research study was used for comparison (animal permission number: ZH272/16). Signal‐to‐noise ratio (SNR) and full width at half maximum (FWHM) were comparable between the investigated dog (SNR, 10; FWHM, 4) and healthy controls (SNR range, 9‐18; median, 14.5; FWHM range, 2.9‐5.9 Hz; median, 4.4 Hz) (Figure 1). Metabolites and their ratios to tCr of the Beagle with LD were considered increased or decreased if they were outside of the range of the healthy Beagle dogs' metabolite concentrations and their ratios to tCr.

Magnetic resonance imaging examination of the investigated dog brain did not identify any abnormalities.

The results of 1HMRS are presented in the Table 1 and Figure 1. Compared to metabolite concentrations found in the healthy controls, the molar concentrations of the sum of glutamate and glutamine [Glx], NAA, and the sum of mI and Gly [mI + Gly] were decreased in the LD dog whereas increased concentrations of tCho and PE were found. Abnormal ratios to tCr were observed for Glx (decreased), tCho (increased), and PE (increased).

**TABLE 1:** Relevant brain metabolites of the Beagle with LD compared with control group

Brain metabolite	Beagle with LD			Healthy controls		
	CRLB (%)	Concentration relative to water	Ratio to tCr	CRLB (%)	Concentration range relative to water	Range of ratio to tCr
tCr	4	7.72	‐	3.08	7.24‐8.96	1.00
mI	9	8.26	1.07	6.5	7.39‐10.46	0.95‐1.31
mI + Gly	5	9.54	1.24	3.33	9.91‐11.50	1.16‐1.49
NAA	6	6.47	0.84	4.75	6.78‐8.06	0.80‐1.03
Glx	11	9.07	1.18	4.17	11.78‐15.79	1.43‐1.92
tCho	5	2.47	0.32	4.17	1.97‐2.40	0.24‐0.32
PE	23	4.06	0.53	38.67	1.23‐3.52	0.16‐0.45

Abbreviations: CRLB, Cramér Rao lower bound; Glx, glutamate‐glutamine complex; Gly, glycine; LD, Lafora disease; mI, myo‐inositol; NAA, *N*‐acetyl‐aspartate; PE, phosphoethanolamine; tCho, total choline; tCr, total creatine.

In the cerebrospinal fluid, cell count, cell types, and protein concentration were within normal limits. The genetic test confirmed LD.

The phenobarbital dosage was adjusted appropriately, but the Beagle still continued to have myoclonus seizures with increased frequency. Therefore, treatment with phenobarbital was continued for generalized tonic‐clonic seizures, and levetiracetam (20 mg/kg PO q8h) was added in an attempt to better control the myoclonus seizures.^1^, ^18^ Additionally, the dog was fed a commercial food to support the nervous system. The owner reported improvement with no additional tonic‐clonic seizures and decreased frequency (approximately 50% according to the owner's observations) of myoclonus seizures.

## 2 DISCUSSION

Proton magnetic resonance spectroscopy is an imaging technique that provides specific biochemical information on numerous intracellular metabolites in a noninvasive way.^6^, ^12^, ^13^ In humans, 1HMRS is used to screen for metabolic abnormalities such as inborn errors of metabolism.^6^, ^12^, ^19^ Proton magnetic resonance spectroscopy findings in dogs with metabolic diseases are sparse.^7^, ^20^, ^21^, ^22^ We were able to detect metabolic changes in the brain of the Beagle dog with LD using 1HMRS despite a lack of abnormalities using conventional MRI. This difference also is common in humans with LD.^14^, ^15^, ^16^ In fact, volumetric brain measurements in humans with LD were not significantly different from these of healthy controls.^14^

*N*‐acetyl‐aspartate is synthetized in neuronal mitochondria and is transported along the axons. Therefore, a normal concentration of NAA indicates neuronal and axonal integrity, and a decrease suggests neuronal damage and loss.^23^ *N*‐acetyl‐aspartate was decreased in the Beagle with LD compared to controls. A decrease of NAA in cerebral cortex, basal nuclei and cerebellum has been detected in studies of humans with LD.^14^, ^15^, ^16^ Glutamate is an excitatory neurotransmitter, and glutamine is its precursor, whereas Gly is an inhibitory neurotransmitter and an *N*‐methyl‐d‐aspartate receptor coagonist.^12^, ^24^ Glutamate and glutamine often are identified as a complex using 1HMRS.^12^ In the Beagle with LD, Glx and Gly + mI were decreased compared to healthy controls. We speculate that the decrement of Glx might be the result of treatment with phenobarbital. In studies of humans, 1HMRS has been performed for the purpose of evaluating γ‐aminobutyric acid as well as glutamate and glutamine concentrations in the brain under the influence of antiepileptic drugs with controversial results.^25^, ^26^ To our knowledge, no 1HMRS investigations of neurotransmitter concentrations in patients treated with phenobarbital have been reported. In human patients suffering from LD, no changes of Glx have been reported, but the majority of these patients were treated with antiepileptic drugs, with 1 patient being treated with phenobarbital.^14^, ^15^, ^16^ Therefore, it is unclear whether pretreatment with phenobarbital influenced the decrease of Glx or if it is a feature of LD in dogs. The signals of Gly and mI are difficult to separate using 1HMRS with a TE of 32 ms measured by 3‐Tesla MRI, but we suspect that Gly contributed more to the decrease of mI + Gly in the investigated LD dog. The mI concentration alone was comparable to that of the control Beagles (Table 1). A decrease of Gly as a consequence of phenobarbital administration has not been described and therefore the Gly concentration in the brain might be decreased as a consequence of LD.

Choline and choline‐containing compounds are important in cell membrane synthesis.^12^ Total choline was increased in the Beagle with LD. Individual brain metabolite analysis with LCModel was suggestive mainly of GPC contribution to increased tCho concentration. In addition, the main peak of PE appears at the same position in the spectrum as the methyl protons of choline‐containing compounds and a reliable estimation of the PE contribution is difficult to make (resulting in high uncertainty in the determined concentration for this metabolite of low concentration). The observed increase in PE therefore could merely reflect the increase in tCho. Phosphoethanolamine is a precursor of cell membrane phospholipids.^27^, ^28^ Glycerophosphocholine, on the other hand, is a compound detected after cell membrane destruction.^29^ In human patients with LD, the ratio of tCho to tCr was significantly increased in the frontal cortex, which was explained by gliosis,^14^, ^15^ but an increase of tCho was not identified in another study.^16^ The increase of tCho in the investigated dog could be associated with neuronal cell membrane destruction, demyelination and, less likely, gliosis. Only minimal gliosis has been found in histopathological examinations of dogs with LD, and the mI concentration (a marker of gliosis) in the investigated dog was within the concentration range of the control group (Table 1).^5^

In a study investigating dogs with hepatic encephalopathy (HE), 1HMRS of the basal nuclei area identified increased concentrations of Glx and decreased concentrations of NAA, tCho, and mI relative to water and tCr compared to controls.^7^ Similarly to the dogs with HE, brain 1HMRS of the Beagle described here showed a decrease of NAA, but, in contrast to dogs diagnosed with HE, Glx ratio to water and tCr was decreased (Figure 1).

Two case reports investigating dogs with the lysosomal storage disease GM2‐gangliosidosis identified a decrease of NAA/tCre and an increase of tCho/tCre in the cerebellar white matter and frontal cortex compared to controls.^20^, ^21^ Additionally, Gly + mI/tCr, and lactate + alanine/tCr ratios in the frontal cortex were increased in 1 of the 2 cases compared with healthy dogs.^20^ A long echo time 1HMRS was performed in the latter case report, which hampers detection of Glx.^20^ Information of the echo time was not available in the other case report.^21^ Decreased NAA/tCre and increased tCho/tCre ratios were common findings in the investigated Beagle dog with LD and the previously described cases. In contrast to the case report describing 1HMRS findings in dog suffering from GM2‐gangliosidosis, mI + Gly/tCr was decreased in the Beagle with LD.

Our case report has several limitations. First, no histopathological examination was performed on the Beagle. Nevertheless, the dog had a typical signalment and clinical signs, and LD was confirmed genetically. Second, only 1 Beagle with LD was investigated, which may not reflect the 1HMRS features in the entire population of dogs suffering from LD. Third, the voxel was positioned in the thalamic area in the Beagle with LD, which precludes direct comparison of investigations of humans with LD, in whom voxels were placed in cerebral cortex, basal ganglia, and cerebellum.^14^, ^15^, ^16^ On the other hand, Lafora bodies are present in thalamic area in humans, dogs, and mice affected by the disease, and the 1HMRS voxels also were positioned in diencephalon in the healthy Beagles, enabling a reliable comparison between findings in the dog with LD and control group.^5^, ^30^, ^31^ In the study investigating dogs with HE, 1HMRS in the thalamic area of 12 healthy Beagles found results comparable to those of our control group.^7^, ^17^

## 3 CONCLUSIONS

Changes of brain metabolites in a Beagle dog with LD were detectable with 1HMRS despite absent abnormalities using conventional MRI. Changes included decreased NAA, Gly + mI, and Glx and increased tCho concentrations. Possibly, 1HMRS spectra of dogs with LD may have features distinct from those of other metabolic diseases. Associations between 1HMRS findings and clinical sign severity in LD remain unknown in dogs, but investigation of such associations should be a future study objective. Proton magnetic resonance spectroscopy might help differentiate between metabolic brain disorders and monitor the effect of treatments in the future.

## CONFLICT OF INTEREST DECLARATION

Authors declare no conflict of interest.

## OFF‐LABEL ANTIMICROBIAL DECLARATION

Authors declare no off‐label use of antimicrobials.

## INSTITUTIONAL ANIMAL CARE AND USE COMMITTEE (IACUC) OR OTHER APPROVAL DECLARATION

Authors declare no IACUC or other approval was needed.

## HUMAN ETHICS APPROVAL DECLARATION

Authors declare human ethics approval was not needed for this study.
